# Transgenic biotechnology in forestry: what a long strange trip it's been

**DOI:** 10.1186/1753-6561-5-S7-I25

**Published:** 2011-09-13

**Authors:** Steven H Strauss

**Affiliations:** 1 Department of Forest Ecosystems and Society, 321 Richardson Hall, Oregon State University, Corvallis, OR, 97331-5752, USA

## 

**Figure 1:**
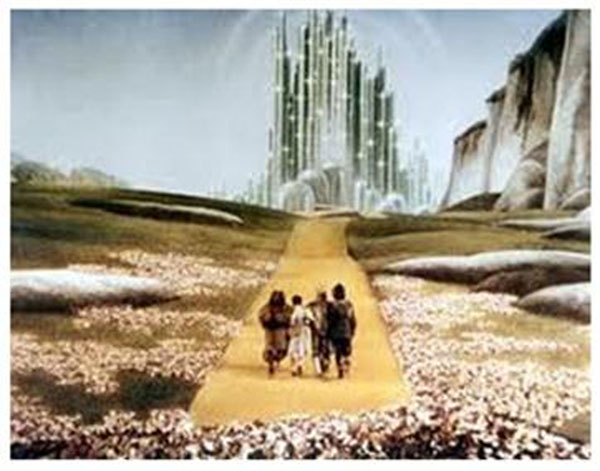
On the road to transgenic Oz in the early days of plant and forest biotechnology.

In the 1980s, when the production of transgenic plants was first demonstrated and was soon followed by production of healthy transgenic poplars in the US and then in Belgium, many of us, including some of the most conservation minded in forestry science, embraced genetic engineering/genetic modification as an important new technology for forestry. We had this sense of following the yellow brick road to Oz. It seemed that we could finally get beyond the narrow limitations of the slow outbreeding, quantitative breeding system, and begin an era of qualitative genetics to complement the dominant quantitative genetic paradigm. Unfortunately, when we got closer to the Wizard of Oz things looked a bit different than on the famed Yellow Brick Road (Figure 1).

What happened? Most important, the technology, and how it was structured, ran into a social and political buzz-saw. The world was becoming richer and more sensitive, both in terms of environment and respect for life. A diversity of social movements were taking place, powered by the new internet, that was uncomfortable with the notion of transgenics and who controlled them, and a number of organizations with resources and power opposed transgenics at every turn. A patent system was put in place that only very large companies or a few wealthy foundations could afford to negotiate. Rich consumers and vulnerable consumers sensed risk and little or no direct benefit to them, and thus also opposed the technology in large numbers. Regulations were pushed through that made the costs of field research, marketing, permits, international trade, liability insurance, and labeling huge barriers to investment and commercial adoption. Nearly the only crops to survive produced huge economic benefits and had to be pushed through the regulatory system by the multinational companies that consumers feel the least trust in.

**Figure 2:**
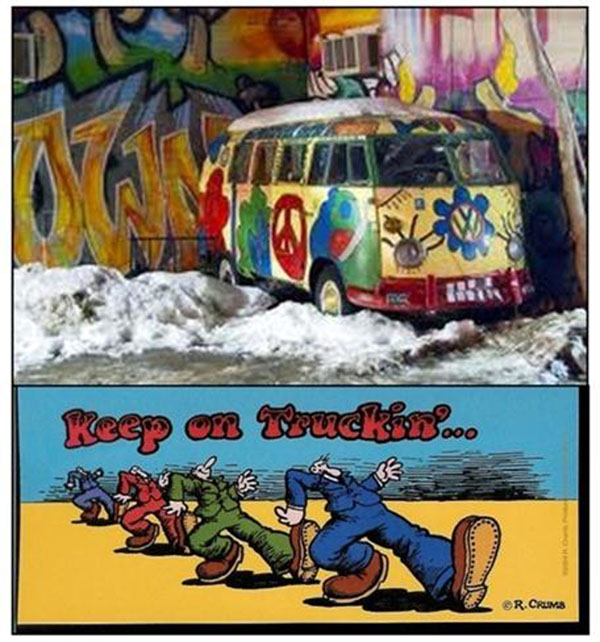
The trip to transgenic Oz was far risker, but also far richer and more informative about ourselves and our society, than we dard to imagine. We will keep on working and innovating with transgenic tools, both on biological and sociopolitical fronts, because our thirst, imagination, and world demands it.

Unfortunately, national and international regulations, once in place, are very hard to change—so this legacy will be with us for many years. Moreover, because the regulations treat all transgenes as hazards until “proven safe,” they pose severe problems for conducting high quality field trials of most types of transgenic forest trees. This is a result of their wild and feral relatives, low level of domestication, and ability to produce pollen and seed that can move over large distances. Thus, it's very hard to integrate transgenic trees into conventional breeding and field testing programs—which is essential for their development and application. Meanwhile, social perspectives and the structure of research funding has led to extraordinary scrutiny and amplification of every possible risk of transgenic trees, usually far out of proportion to its significance compared to conventional agriculture and forestry breeding. Thus, the trip has felt much more like a certain Electric Kool-Aid Test (Figure 2), than a normal technological progression, and not at all like the start of the trip to Oz.

On the science side, despite our early successes, when we finally got to meet the Wizard, there was also a number of revelations that gave us pause about the robustness of transgenic tree biotechnology, and most remain significant to this day. 1) It was much harder to transform/regenerate most commercially important forest tree species/genotypes than we had imagined, and very little in the way of public research funding has been directed to overcome this in a meaningful way. 2) There was a great deal of hype about some of the early successes, such as in the area of growth rate improvement and lignin modification, with insufficient field trials to judge their true merit. Many have not proven themselves, but the results are often hidden by business confidentiality, academic self-interest, and a paucity of high quality field research 3) Beyond 35S-type overexpression for traits like herbicide and Bt-insect resistance, the tools in place for control of gene expression were found to be coarse, unpredictable, and imprecise, likely due to lack of control of insertion location, chromatin state, and poor understanding of RNAi/siRNA mechanisms. 4) While genetic containment would solve a lot of social and regulatory problems, due to high social concerns, short term funding, strict regulations over field trials, and immature technology, it's unclear if we can attain, in the near to middle term, the nearly perfect level of predictable, field-validated containment that appears to be required. 5) The extraordinary advances in genomics and phenomics in conventional breeding, combined with the high costs of regulations, are pushing transgenic applications to focus on the most new, novel, and valuable applications that cannot be attained by conventional or genome-assisted breeding—which are also those for which attaining regulatory approval will be most difficult. 6) Meaningful, field-based ecological studies of fitness and non-target effects, especially of pest-resistant, stress-resistant, bioindustrial, and bioremediation types of transgenic trees, have not been conducted—leaving a wide swath for models and speculations of severe ecological impact that are likely to be vastly overstated—but push regulations toward ever greater stringency. In sum, a great deal of fundamental science, from the genic to the ecological, and technology development to efficiently identify and transfer genes and pathways, remains to be done by the next generation of scientists and practitioners.

Propelled by the ongoing successes of some pioneering companies, I sense that transgenic forest biotechnology, though facing great difficult challenges at present, will “keep on trucking” in the parlance of “The Trip” (Figure 2). This will be driven by science and technology successes that continue at an impressive rate; urgency in finding solutions in the face of severe global economic and environmental problems; and the human thirst for novelty, innovation, and truth.

